# Solving the Static Resource-Allocation Problem in SDM-EONs via a Node-Type ILP Model

**DOI:** 10.3390/s22249710

**Published:** 2022-12-11

**Authors:** Jiading Wang, Sibo Chen, Qian Wu, Yiliu Tan, Maiko Shigeno

**Affiliations:** 1 Graduate School of Systems and Information Engineering, University of Tsukuba, Tsukuba 305-8573, Japan; 2 Department of Applied Informatics, Faculty of Science and Engineering, Hosei University, Tokyo 102-8160, Japan

**Keywords:** SDM-EONs, static planning problem, resource allocation, ILP model, space lane change

## Abstract

Space division multiplexing elastic optical networks (SDM-EONs) are one of the most promising network architectures that satisfy the rapidly growing traffic of the internet. However, different from traditional wavelength division multiplexing (WDM)-based networks, the problems of resource allocation become more complicated because SDM-EONs have smaller spectrum granularity and have to consider several novel network resources, such as modulation formats and spatial dimensions. In this work, we propose an integer linear programming (ILP) model without space lane change (SLC) that provides theoretically exact solutions for the problem of routing, modulation format, space, and spectrum assignment (RMSSA). Moreover, to more efficiently solve our model which is difficult to solve directly, we propose three exact algorithms based on model decomposition and evaluate their performance via simulation experiments, and we find that two of our exact algorithms can solve the model effectively in small-scale instances.

## 1. Introduction

In recent years, the rapid growth of global network traffic has been the driving force behind innovations in optical network technologies and architectures. Compared to fixed-grid wavelength division multiplexing (WDM)-based networks, known as wavelength-switched optical networks (WSONs), flexible-grid elastic optical networks (EONs) [1,2] are able to transmit connection requests with higher spectrum efficiency. While EONs can provide smaller spectrum granularity and increase network capacity to some extent, the increase in network capacity that they can provide is limited because of the nonlinear Shannon limit of single-mode fibers (SMFs). To overcome this limitation, space division multiplexing (SDM) [3,4] was introduced as one of the promising solutions for EONs, and these new network architectures are called SDM-EONs. In SDM-EONs, nodes are connected by fibers with multiple spatial dimensions, such as fiber bundles (FBs), multicore fibers (MCFs) and few-mode fibers (FMFs) [5]. By introducing multiple spatial dimensions in this way, a significant increase in optical network capacity can be achieved.

### 1.1. Problems of Resource Allocation

For the design of optical transport networks (OTNs), the mathematical optimization problem considering routing, spectrum, and other network resources is called the resource allocation problem for OTNs. Solving the resource allocation problem is significant for reducing network cost and saving energy.

In fixed-grid WDM-based WSONs, the resource allocation problem is referred to as the routing and wavelength assignment (RWA) problem. Specifically, finding a light path for any connection request in an optical network that is established by occupying the same wavelength on all the links it passes through is called the wavelength continuity restriction; in addition, a single wavelength can be used once at most, which is called the wavelength nonoverlapping restriction. This problem has been proven to be NP-hard and was investigated in many previous works [6,7].

In 2009, Jinno et al. [1] proposed the SLICE architecture, which drove the first evolution of optical network architectures from WSONs to EONs. Due to the employment of bandwidth-variable optical transceivers (BVTs) and bandwidth-variable optical cross-connects (BV-OXCs), EONs can use super channels (SpChs) consisting of different numbers of contiguous frequency slots (FSs) without guard-band (GB) intervals to flexibly establish light paths based on the traffic required for connection requests. Compared to WSONs, EONs achieve higher spectrum efficiency and more flexible transmission. However, the resource-allocation problem is more complex than the RWA problem in WSONs due to the additional restrictions that EONs imposed on the establishment of light paths, i.e., the FSs assigned to the light paths must be contiguous in the spectral domain, which is called the FSs contiguity restriction. Such a problem is well-known as the routing and spectrum assignment (RSA) problem. Similar to the RWA problem, the RSA problem was also proven to be NP-hard [8,9]. In addition, when considering multiple modulation formats, EONs support using distance adaptive transmission (DAT) to select the modulation formats, i.e., selecting appropriate modulation formats for optical signals depending on the length of the light paths [10]. In this context, the RSA problem turns into the routing, modulation format and spectrum assignment (RMSA) problem [11,12].

In SDM-EONs, because of the introduction of multiple spatial dimensions, light-path selection needs to consider the assignment of space lanes (SLs), which makes the resource-allocation problem more complex. Such a resource-allocation problem is the routing, space and spectrum assignment (RSSA) problem [5]. Similarly, the RSSA problem is complexed into a routing, modulation format, space, and spectrum assignment (RMSSA) problem when multiple modulation formats are considered.

### 1.2. Related Works

Resource-allocation problems in optical networks, such as RWA in WSON, RSA in EONs, and RSSA in SDM-EONs, can be divided into static (e.g., [5,9,10,11,12,13,14,15,16,17,18,19,20,21,22,23,24,25,26,27,28,29]) and dynamic scenarios (e.g., [21,30,31,32,33,34,35]). In static scenarios, all connection requests are known in advance, and the objectives are usually to determine the network design required to accommodate all known connection requests or to minimize the amount of resources required for various sets of known connection requests. In contrast, in dynamic scenarios, we deal with connection requests that occur and disappear based on the passage of time. We typically simulate actual connection situations and study how to design the network and distribute connection requests so that connection requests are served in real time [12].

To solve problems such as RSA or RSSA, mathematical optimization techniques are commonly adopted. These techniques can be divided into two approaches. When we formulate the problem as an integer linear programming (ILP) problem, we can solve it exactly using ILP solvers [11,23]. This approach can be adopted for static scenarios that require exact optimal solutions with sufficient time for investigation. In contrast, using heuristic algorithms enables finding better solutions within a relatively short time (e.g., [19,21,24]). In particular, in dynamic scenarios where fast and reasonable resource allocation solutions are needed but not necessarily the exact optimal solutions, heuristic algorithms are usually adopted instead of ILP models, which find the exact solutions.

ILP models can be classified into path-type models (e.g., [9,16]), which select paths from a set of candidate paths, and node-type models (e.g., [21,22]), which consider all available paths according to the modeling approaches on routing [12]. Notably, for a path-type model that employs a set of candidate paths, the solutions obtained by the model are exact if the set of candidate paths considers all available paths, which we call all-path-type; the solutions obtained by the model are not necessarily exact if the set of candidate paths includes only a part of the available paths, which we call the k-path-type. To the best of our knowledge, the vast majority of previous works employing the path-type models used the k-path-type. Moreover, for RMSA or RMSSA problems that consider multiple modulation formats, ILP models are correspondingly complicated.

In addition, space lane change (SLC) is a non-negligible transmission technique in SDM-EONs. Specifically, with the adoption of SLC technology, connection requests can disregard spatial continuity restrictions and use different spatial dimensions on different links of a light path. Although SLC can further increase the routing flexibility at the same spatial switching granularity to enable higher spectral efficiency, it consumes higher equipment costs due to its deployment of wavelength selective switches (WSSs) with higher port counts. Several papers evaluated the improvements in spectrum allocation that SLC brings in dynamic and static scenarios. In dynamic scenarios, the consideration of SLC can lead to a 7% to 14% improvement of network throughput, which was evaluated in Refs. [34,35]. In static scenarios, the savings in spectrum resources that SLC can bring (0.1% to 3.1%) are negligible compared to its equipment cost, which was addressed in Ref. [25].

### 1.3. Contributions

To the best of our knowledge, in the static scenarios of SDM-EONs, there is not yet any work that solves the RMSSA problem, which involves multiple modulation formats by formulating node-type ILP models. In our work, considering the limitations of the role of SLC in static scenarios, a novel non-SLC node-type ILP model considering all three types of SpChs is proposed, and several computational methods to speed up the model are discussed. Furthermore, this model can provide better solutions than the k-path-type model in Ref. [11]. Table 1 shows the difference between our work and previous works that formulated ILP models to describe the resource-allocation problems for OTNs.

The rest of this paper is organized into five sections. In Section 2, we introduce the related background knowledge and techniques of SDM-EONs, such as SpChs and switching paradigms. In Section 3, we depict our non-SLC node-type ILP model for solving the RMSSA problem with the objective of minimizing the maximum FS index and propose three exact algorithms that can accelerate the model. In Section 4, we compare the three exact algorithms for solving our model and verify the effectiveness of the three algorithms via the lower bounds of our model and the results of a heuristic algorithm. In addition, we compare our model with the previous k-path-type one in Ref. [11] via simulation experiments. Finally, in Section 5, we conclude this paper.

## 2. Background and Assumptions

In this section, we briefly introduce some of the key transmission techniques for SDM-EONs that are considered in this work.

### 2.1. Types of Super Channels

**Figure 1:**
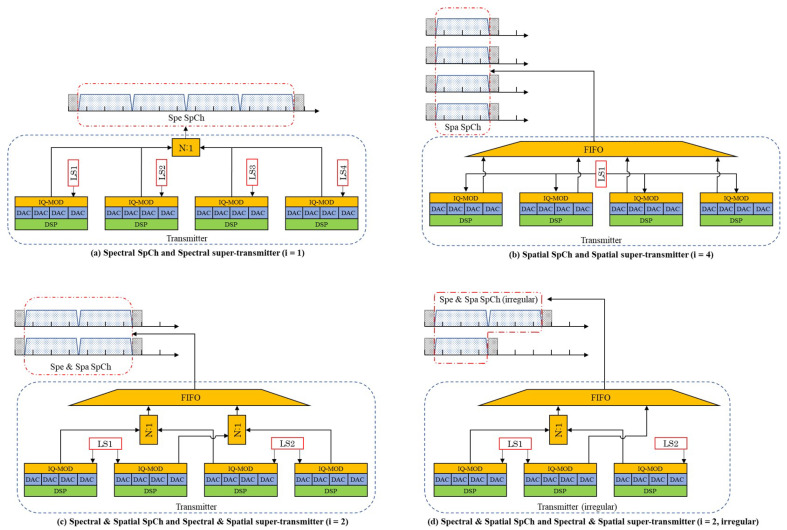
Examples of Spe SpCh, Spa SpCh, Spe and Spa SpCh, and their corresponding transmitters at different spatial switching granularities *i*, which are equal to 1, 2 and 4. (**a**) Spectral SpCh and Spectral super-transmitter at i=1; (**b**) Spatial SpCh and Spatial super-transmitter at i=4; (**c**) Spectral and Spatial SpCh and Spectral and Spatial super-transmitter at i=2; (**d**) Irregular Spectral and Spatial SpCh and Spectral and Spatial super-transmitter at i=2. DSP: digital signal processing; DAC: digital-to-analog converter; IQ-MOD: in-phase and quadrature modulator; LS: laser source; N:1: coupler; FIFO: fan-in/fan-out.

Three types of superchannels with different spatial switching granularities have been proposed in SDM-EONs, and they are the spectral superchannel (Spe SpCh), the spatial superchannel (Spa SpCh), and the spectral and spatial superchannel (Spe and Spa SpCh), as shown in Figure 1 [5]. Here, we assume that single-mode fibers are employed, and the spatial dimension of an SDM-EON is 4 (i.e., the network is interconnected via 4-core MCFs or 4-fiber SMFBs). Then, the superchannels with spatial switching granularities *i*, which are equal to 1, 2 and 4, correspond to Spe SpCh, Spe and Spa SpCh, and Spa SpCh, respectively.

- As shown in Figure 1a, a Spe SpCh is composed of several continuous optical carriers (OCs) that are generated by several continuous single-carrier transmitters. Switching GBs exist only between adjacent SpChs, and there are no switching GBs between two adjacent OCs in the same SpCh. Since a Spe SpCh takes only two switching GBs at two sides of the SpCh (i.e., 1FS), it has the highest spectral efficiency. However, a Spe SpCh containing four OCs, as in the example in the figure, uses four independent laser sources; hence, it is the most costly.
- Figure 1b illustrates an Spa SpCh containing four OCs, and it is generated by a 4×1 joint transmitter and uses a common laser source in different spatial dimensions. Thus, it has the lowest cost, although it has the lowest spectral efficiency.
- A Spe and Spa SpCh containing four OCs is shown in Figure 1c, and it is generated by a 2×2 fractional joint transmitter. OCs allocated on the same spectrum can share a common laser at the transmitter. It is obvious that the Spe and Spa SpCh is a hybrid SpCh that provides intermediate cost and spectral efficiency. Figure 1d shows an irregular Spe and Spa SpCh containing three OCs, which is not considered in this work.

### 2.2. Switching Paradigms for Different Super Channels

There are three switching paradigms, independent switching (In-Sw), fractional joint switching (FrJ-Sw) and joint switching (J-Sw), corresponding to the Spe SpCh, Spa SpCh, and Spe and Spa SpCh, respectively. In-Sw and FrJ-Sw can each be subdivided into two types based on whether or not they support SLC [35].

**Figure 2:**
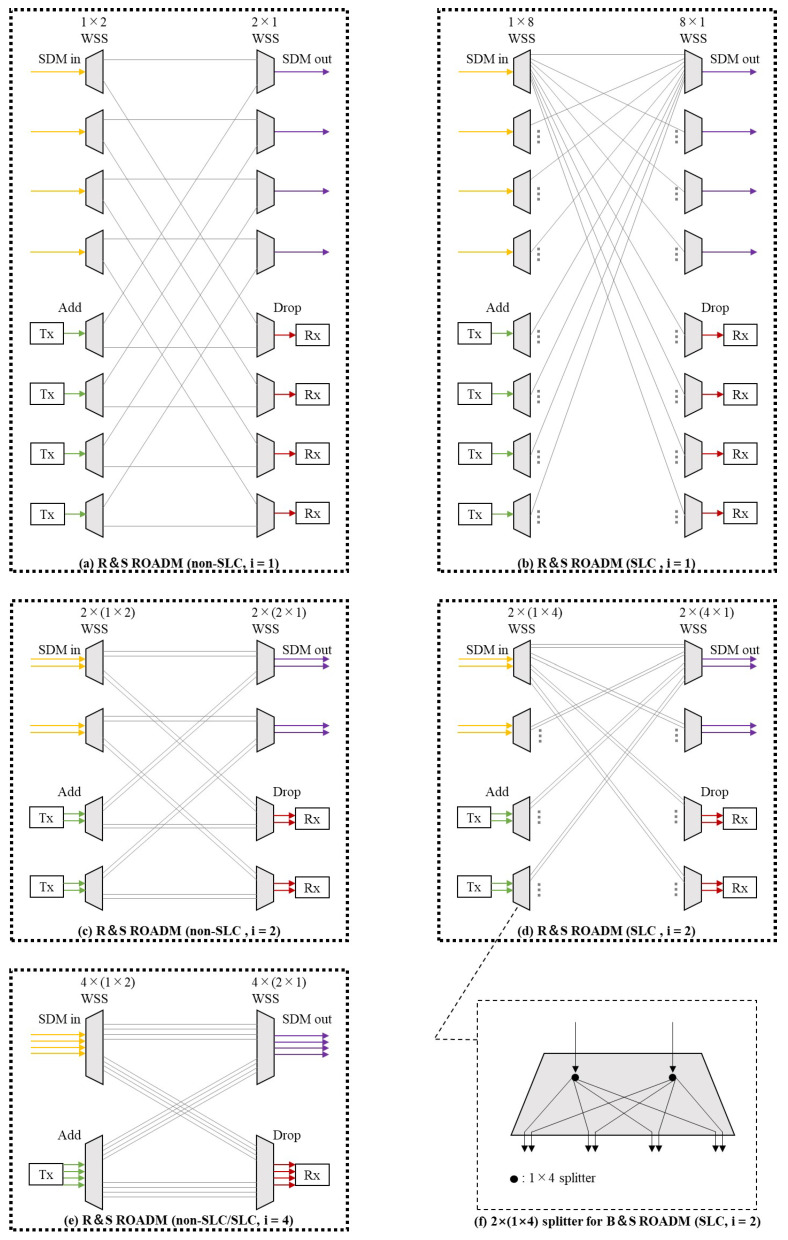
ROADM architectures for various switching paradigms at spatial switching granularities *i*, which are equal to 1, 2 and 4, without and with SLC. (**a**) R and S ROADM for In-Sw without SLC; (**b**) R and S ROADM for In-Sw with SLC; (**c**) R and S ROADM for FrJ-Sw without SLC; (**d**) R and S ROADM for Frj-Sw without SLC; (**e**) R and S ROADM for J-Sw with and without SLC; (**f**) 2×(1×4) splitter for B and S ROADM. R and S ROADM: route-and-select reconfigurable optical add-drop multiplexer; B and S ROADM: broadcast-and-select reconfigurable optical add-drop multiplexer; Tx: transmitter; Rx: receiver.

Figure 2 shows the architectures of the reconfigurable optical add-drop multiplexers (ROADMs) corresponding to the various switching paradigms with and without SLC. The ROADMs in the figure are all at the 2-degree (i.e., SDM in and SDM out) intermediate nodes in a network that has four spatial dimensions on each link.

- Figure 2a,b illustrate the architectures of the route-and-select (R and S) ROADMs corresponding to In-Sw without and with SLC, respectively. In this instance, the spatial switching granularity *i* is equal to 1. It is observed that the ROADM structure supporting SLC has a higher port count per WSS, resulting in higher costs. As we stated in Section 1.2, the spectrum savings that SLC can bring in the RMSSA problem in static scenarios are very limited; therefore, in this work, we discuss the non-SLC case.
- Figure 2c,d illustrate the architectures of the R and S ROADMs corresponding to FrJ-Sw without and with SLC, respectively. In this instance, the spatial switching granularity *i* is equal to two.
- Figure 2e shows the architecture of the R and S ROADM corresponding to J-Sw with and without SLC. In this instance, the spatial switching granularity *i* is equal to 4. Since there is no spatial dimension group that can be changed when the connection requests pass through the ROADMs, the structures of the ROADMs are the same for the SLC and non-SLC cases when the switching paradigm is J-Sw. In addition, it should be noted that if all WSSs on the LHSs of Figure 2a–e are replaced by splitters with the corresponding dimensions (e.g., Figure 2f for Figure 2d), the R and S ROADMs in the figures will change to broadcast-and-select (B and S) ROADMs.

## 3. ILP Model and Algorithms for the RMSSA Problem

In this section, we construct a node-type ILP model for the static non-SLC RMSSA problem (referred to as the non-SLC node-type ILP model) with arbitrary spatial switching granularity and develop three algorithms based on the model, which are used to solve the model exactly. The notations used in the ILP model are shown in Table 2.

To construct a set of available modulation levels MF, we assign modulation levels from small to large for different modulation formats from low to high spectral efficiencies. For instance, we can assign modulation levels 1, 2, 3, and 4 in MF corresponding to the modulation formats BPSK, QPSK, 8-QAM, and 16-QAM with spectral efficiencies of 1, 2, 3, and 4 [b/s/Hz], respectively [10]. Thus, in the node-type ILP model, the number of FSs $n_{r}^{mi}$ can be calculated by Equation (1). In this paper, we assume that the spectrum grid (i.e., $W_{FS}$) is 12.5 GHz based on the ITU-T standard G.694.1, the transceiver transmits/receives an OC with 37.5 GHz (i.e., $W_{OC}$) bandwidth at a fixed 32 Gbaud baud rate, and the bandwidth of the switching GB on each side of an SpCh (i.e., $W_{GB}$) is 6.25 GHz.

(1) $$n_{r}^{mi}=\left\lceil \frac{\left\lceil \lceil \frac{T_{r}}{m\cdot t_{oc}}\rceil /i\right\rceil \cdot W_{OC}+2W_{GB}}{W_{FS}}\right\rceil$$

### 3.1. Non-SLC Node-Type ILP Model

A non-SLC node-type ILP model for any spatial switching granularity *i* can be formulated as follows.

Objective

(2) $$\mathrm{minimize}\;F_{max}$$

It is subject to

(3) $$\begin{array}{c} \sum_{e\in \sigma_{v}^{+}}x_{r}^{eg}-\sum_{e\in \sigma_{v}^{-}}x_{r}^{eg}=\left\{\begin{array}{cc} 1 & \mathrm{if}\;v=s_{r}\\ -1 & \mathrm{if}\;v=d_{r}\\ 0 & \mathrm{otherwise} \end{array}\right.\\ \forall v\in V,r\in R,g\in G_{i} \end{array}$$

(4) $$\sum_{e\in \sigma_{v}^{-}}\sum_{g\in G_{i}}x_{r}^{eg}\leq 1,\;\forall v\in V,r\in R$$

(5) $$\sum_{e\in \sigma_{v}^{+}}\sum_{g\in G_{i}}x_{r}^{eg}\leq 1,\;\forall v\in V,r\in R$$

(6) $$h_{r}\geq \sum_{e\in E}\sum_{g\in G_{i}}l^{e}x_{r}^{eg},\;\forall r\in R$$

(7) $$\sum_{m\in MF}a^{m}u_{r}^{m}\geq h_{r},\;\forall r\in R$$

(8) $$\sum_{m\in MF}u_{r}^{m}=1,\;\forall r\in R$$

(9) $$o_{rr^{\prime }}+o_{r^{\prime }r}=1,\;\forall r,r^{\prime }\in R:r\neq r^{\prime }$$

(10) $$\begin{array}{c} f_{r}+\sum_{m\in MF}n_{r}^{mi}u_{r}^{m}\leq f_{r^{\prime }}+M\left(3-x_{r}^{eg}-x_{r^{\prime }}^{eg}-o_{rr^{\prime }}\right)\\ \forall r,r^{\prime }\in R,e\in E,g\in G_{i}:r\neq r^{\prime } \end{array}$$

(11) $$f_{r}+\sum_{m\in MF}n_{r}^{mi}u_{r}^{m}-1\leq F_{max},\;\forall r\in R$$

Our objective is to minimize the maximum index of the required FSs $F_{max}$ in the network topology, as shown in Equation (2). Equations (3)–(5) ensure that only one path and one spatial dimension group are used for each connection request. Specifically, Equation (3) uses the law of flow conservation. If the light path transmitting the connection request, *r* uses the spatial dimension group *g* on link *e*, then the LHS of Equation (3) will equal 1 at the source node $s_{r}$ and −1 at the destination node $d_{r}$, and it will equal 0 at inter nodes on the light path and at nodes not on the light path. Equations (4) and (5) enable there to be no cycle in the light path. Equations (6)–(8) represent that only one modulation level, which determines the length of the path, can be selected for any connection request. Equations (9) and (10) prevent the overlapping of the spectrum used by different connection requests. Equation (11) expresses the calculation method of $F_{max}$.

Since the number of variables and constraints of the model are proportional to ${\left|R\right|}^{2}$, |E| and $|G_{i}|$, the model will become increasingly difficult to solve as the number of connection requests, spatial dimensions and scale of the network topology increase. Hence, for large-scale instances, such as dealing with a large number of connection requests in a network topology with a large number of nodes, links and spatial dimensions, the model may be difficult to solve with ILP solvers. Actually, in our preexperiments that included a small 6-node network, the model was difficult to solve directly when there were more than 100 connection requests in the network, which obviously needs to be improved. Therefore, in the next four subsections, we address three model decomposition algorithms named the direct model decomposition (DMD), all-SLC model decomposition (ASLC-MD), and semi-SLC model decomposition (SSLC-MD) algorithms for enabling the ILP solvers to solve the model more efficiently.

### 3.2. Direct Model Decomposition (DMD) Algorithm

Node-type models are difficult to solve exactly with ILP solvers since the default initial bounds provided by ILP solvers are often of low quality. By first solving the relaxation model, which is obtained by decomposing the original model, it is possible to obtain better initial bounds to speed up solving the original model [9,26].

We decompose the original RMSSA model in SDM-EONs (i.e., the non-SLC node-type model in Section 3.1 that solves the RMSSA problem) into RMSA model (the object is Equation (12) to satisfy Equations (3)–(8) and (13)–(15)) and the spectrum assignment (SA) model (the object is Equation (16) to satisfy Equations (9) and (17)–(20)). Here, $F_{max}^{lb}$ and $F_{max}^{ub}$ are non-negative integer variables that represent the maximum index of the required FSs in the RMSA and SA models, respectively, and their values obtained by solving these two decomposition models (i.e., the RMSA and SA models) can be used as the lower and upper bound values for the original RMSSA model separately. The notation $b_{r}^{meg}$ indicates a binary variable that is equal to 1 if connection request *r* uses modulation level *m* and spatial dimension group *g* on link *e* and 0 otherwise. $m_{r}^{out}$ denotes the selected modulation level for serving connection request *r* obtained by solving the RMSA model.

(12) $$\mathrm{minimize}\;F_{max}^{lb}$$

(13) $$b_{r}^{meg}\leq \frac{1}{2}\cdot \left(u_{r}^{m}+x_{r}^{eg}\right),\;\forall r\in R,m\in MF,e\in E,g\in G_{i}$$

(14) $$b_{r}^{meg}\geq u_{r}^{m}+x_{r}^{eg}-1,\;\forall r\in R,m\in MF,e\in E,g\in G_{i}$$

(15) $$\sum_{r\in R}\sum_{m\in MF}b_{r}^{meg}n_{r}^{mi}-1\leq F_{max}^{lb},\;\forall e\in E,g\in G_{i}$$

(16) $$\mathrm{minimize}\;F_{max}^{ub}$$

(17) $$F_{max}^{ub}\geq F_{max}^{lb}$$

(18) $$F_{max}^{ub}\geq f_{r}+n_{r}^{m_{r}^{out}}-1,\;\forall r\in R$$

(19) $$\begin{array}{c} f_{r}+n_{r}^{m_{r}^{out}i}\leq f_{r^{\prime }}+M\cdot \left(1-o_{rr^{\prime }}\right)\\ \forall r,r^{\prime }\in R:r\neq r^{\prime } \end{array}$$

(20) $$m_{r}^{out}=\left\{m\in MF:r\in R,e\in E,g\in G_{i}|b_{r}^{meg}=1\right\}$$

**Figure 3:**
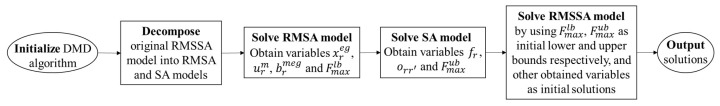
Framework of the DMD algorithm.

As shown in Figure 3, by solving the RMSA model first, we can take the obtained destination function value as the initial lower bound for the original model. After that, we substitute the solutions obtained in the RMSA model as known parameters into the SA model and solve it. Finally, the value of the objective function obtained by solving the SA model is used as the initial upper bound for the original model, and the solutions of the RMSA and SA models are substituted into the original model as the initial solutions to solve it.

### 3.3. All-SLC Model Decomposition (ASLC-MD) Algorithm

If we relax the restrictions related to the spatial continuity of the non-SLC node-type model in Section 3.1 (i.e., Equation (3), the model will be changed to a node-type model that supports SLC. In this case, the formulation related to routing and space using the law of flow conservation in the SLC node-type model will become Equation (21) below.

(21) $$\begin{array}{c} \sum_{e\in \sigma_{v}^{-}}\sum_{g\in G_{i}}x_{r}^{eg}-\sum_{e\in \sigma_{v}^{+}}\sum_{g\in G_{i}}x_{r}^{eg}=\left\{\begin{array}{cc} 1 & \mathrm{if}\;v=s_{r}\\ -1 & \mathrm{if}\;v=d_{r}\\ 0 & \mathrm{otherwise} \end{array}\right.\\ \forall v\in V,r\in R \end{array}$$

Since the number of constraints in the SLC model in Equation (21) is reduced in comparison with the non-SLC model in Equation (3), the time required to solve the SLC node-type model may be shorter than that required for the non-SLC model.

As we mentioned in Section 1.2, from the statements and experimental results in Ref. [25], the effect of the spectrum savings from SLC is negligible in the static scenario. Therefore, we can equate the RMSSA model in Section 3.1 to the SLC-RMSSA model with Equations (2), (4)–(11), and (21)–(24) if the value of its minor objective function (i.e., Equation (22)) is equal to 0. Here, $SLC_{r}^{v}$ denotes a binary variable that is equal to 1 if connection request *r* changes the space lane used in the former link when passing through node *v* and 0 otherwise.

Major objective:

The major objective function aims to minimize the maximum index of the required FSs $F_{max}$ and is the same as the objective function of the non-SLC node-type model in Section 3.1 (i.e., Equation (2)).

Minor objective:

(22) $$\mathrm{minimize}\;\sum_{v\in V}\sum_{r\in R}SLC_{r}^{v}$$

This objective is subject to

(23) $$\begin{array}{c} \sum_{e\in \sigma_{v}^{+}}x_{r}^{eg}-\sum_{e\in \sigma_{v}^{-}}x_{r}^{eg}\leq SLC_{r}^{v}\\ \forall v\in V,r\in R,g\in G_{i}:v\neq s_{r},v\neq d_{r} \end{array}$$

(24) $$\begin{array}{c} \sum_{e\in \sigma_{v}^{-}}x_{r}^{eg}-\sum_{e\in \sigma_{v}^{+}}x_{r}^{eg}\leq SLC_{r}^{v}\\ \forall v\in V,r\in R,g\in G_{i}:v\neq s_{r},v\neq d_{r} \end{array}$$

**Figure 4:**
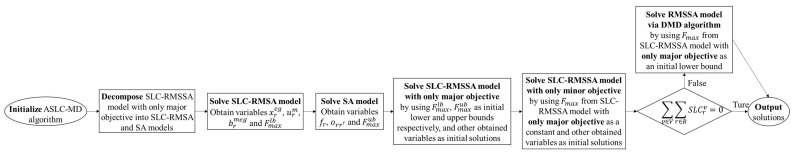
Framework of the ASLC-MD algorithm.

As shown in Figure 4, the ASLC-MD algorithm is actually the process of decomposing and solving the SLC-RMSSA model with major and minor objective functions. First, we address an SLC-RMSSA model with only the major objective, which consists of Equations (2), (4)–(11) and (21). This model is decomposed into the SLC-RMSA and SA models (the SA model is independent of whether SLC is supported or not since it does not include the selection of spatial dimensions). Similar to the DMD algorithm in Section 3.2, we solve the SLC-RMSA and SA models in turn and confer the solutions of the SLC-RMSA and SA models as initial solutions to the SLC-RMSSA model with only the major objective. Different from the DMD algorithm, after that, we address an SLC-RMSSA model with only the minor objective, which consists of Equations (4)–(11) and (21)–(24). The value of $F_{max}$ obtained by solving the SLC-RMSSA model with only the major objective is fixed, and the other variables are assigned to the SLC-RMSSA model with only the minor objective as the initial solution. If the SCL number $\sum_{v\in V}\sum_{r\in R}SLC_{r}^{v}$ is equal to 0 upon solving this model, the solutions of the model are output; otherwise, $F_{max}$ will be brought into the DMD algorithm as an initial lower bound to solve the original RMSSA model.

### 3.4. Semi-SLC Model Decomposition (SSLC-MD) Algorithm

The ASLC-MD algorithm minimizes the SLC number after solving the SLC-RMSSA model, and the step that minimizes the SLC number requires much time because of the high complexity of the SLC-RMSSA model. Therefore, we consider a semi-SLC model decomposition (SSLC-MD) algorithm, which minimizes the SLC number after solving the decomposed SLC-RMSA model to ensure that a light path without SLC is obtained.

**Figure 5:**
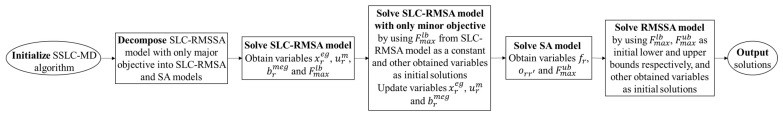
Framework of the SSLC-MD algorithm.

The framework of the SSLC-MD algorithm is shown in Figure 5. Different from the ASLC-MD algorithm, the SSLC-MD algorithm supports SLC only in the RMSA model, while the RMSSA model is the original non-SLC node-type model. The SSLC-MD algorithm minimizes the SLC number (i.e., solving the SLC-RMSA model with only a minor objective) after solving the SLC-RMSA model (i.e., minimizing the maximum index of the required FSs). Since the FSs are not yet assigned in the SLC-RMSA model, whether SLC is considered does not have an impact on the maximum index of the required FSs (i.e., the SLC number in the SLC-RMSA model with SLC can converge to 0).

### 3.5. Analysis for Scales of the Models

The variables and constraints in each model mentioned in Section 3 are shown in Table 3. RMSSA and RMSA indicate the original model proposed in Section 3.1 (i.e., Non-SLC node-type ILP model) and the relaxation model employed by the DMD algorithm in Section 3.2, respectively. SLC-RMSSA and SLC-RMSA denote the node-type ILP model supporting SLC and the relaxation model supporting SLC, respectively, which are employed in the algorithms ASLC-MD in Section 3.3 and SSLC-MD in Section 3.4. SA is the model considering only the spectrum assignment which is used in Section 3.2, Section 3.3 and Section 3.4.

Analyzing the number of variables and constraints for each model, the following can be derived.

(1) The number of connection requests |R| affects RMSSA and SLC-RMSSA significantly because the numbers of variables and constraints in these two models are related to the square of the number of connection requests ${\left|R\right|}^{2}$.
(2) The numbers of variables and constraints of the models RMSA, SLC-RMSA, and SA used in the decomposition algorithms are not affected by the square of the number of connection requests. Thus, it is expected that the decomposition algorithms effectively reduce the computation time compared to solving the original model directly.
(3) SLC-RMSSA and SLC-RMSA show a reduction in the number of constraints (from $O(|R|\cdot |V|\cdot |G_{i}|)$ to O(|R|·|V|)) compared to RMSSA and RMSA. Due to the effect of ${\left|R\right|}^{2}$, the impact on the computation time caused by the change may be negligible in SLC-RMSSA and RMSSA. However, in SLC-RMSA and RMSA, when constraints whose number is $O(|R|\cdot |V|\cdot |G_{i}|)$ (i.e., Equation (3) in Section 3.1, which uses the law of flow conservation) are abundantly present, the change has the potential to reduce the computation time of the model.

## 4. Simulation and Numerical Results

In this section, we first verify the effectiveness of the three algorithms proposed in Section 3 via lower bounds of our model and results of a first-fit greedy (FF-G) algorithm [14].

### 4.1. Environmental Parameters and Assumptions

**Figure 6:**
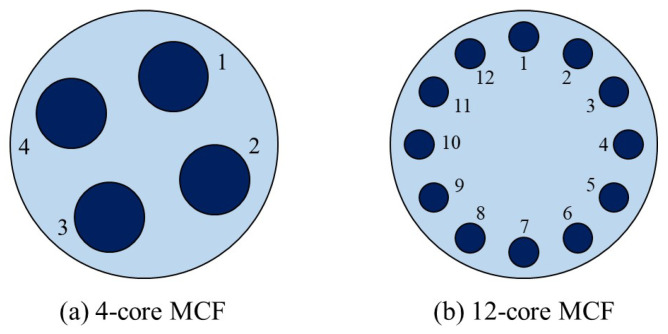
The MCFs considered in the simulation experiments.

We perform the three algorithms above in a 6-node 18-directed link N6S9 network [12]. In addition, we compare our model with the k-path-type model from the previous work [11] in a 14-node 42-directed link NSF network [36]. As shown in Figure 6, we consider several cases, where each link uses the 4-core MCF [37] or the 12-core MCF [38] to connect the network in the simulation experiments.

Based on the ITU-T standard G.694.1, the total number of FSs that each link has (i.e., |F|) is set to 320 (i.e., 12.5 GHz per FS at the C-band with a 4 THz bandwidth) [39]. The number of modulation levels |MF| is set to 4. The numbers 1, 2, 3, and 4 indicate the modulation formats double polarization (DP)-BPSK, DP-QPSK, DP-8QAM, and DP-16QAM, respectively. Each OC is generated by a transceiver, which can support 50 Gbps via DP-BPSK under the 32 Gbaud symbol rate containing 7 GBaud (approximately 20%) for forward error correction (FEC) overhead [40] and occupying in total 37.5 GHz spectrum (i.e., 3 FSs) [41]. Therefore, the supportable bit rates per OC for the modulation formats DP-BPSK, DP-QPSK, DP-8QAM and DP-16QAM are set to 50, 100, 150 and 200 Gbps, respectively.

For the maximum transmission distances (km) of the considered modulation formats above, we consider that they are mainly driven by two factors: a) the optical signal-to-noise ratio (OSNR), and b) the inter-core crosstalk (XT) of MCFs. As shown in Table 4, these physical features of the two types of MCFs we mentioned above are used to calculate the maximum transmission distances for different modulation formats. The parameters *k*, Λ, β and γ represent the coupling coefficient, core pitch, propagation constant, and bend radius, respectively. It should be noted that the coupling coefficients *k* are calculated according to Ref. [42]. In the coherent systems, the maximum transmission distances of different modulation formats are bounded by OSNR, and can be estimated by the Gaussian Noise model of nonlinear interference [43]. The XT of a connection request in MCFs after *D* km can be calculated by Equation (25).

(25) $$XT\left(D\right)=\frac{C-C\cdot exp\{-2(C+1)uD\}}{1+C\cdot exp\{-2(C+1)uD\}},\;where\;u=\frac{2k^{2}\gamma }{\beta \Lambda }$$

In Equation (25), *C* represents the number of adjacent cores of the core transmitting the current connection request. We assume that the thresholds of XT ($XT_{m}^{thre}$) for modulation formats DP-BPSK, DP-QPSK, DP-8QAM, and DP-16QAM are −14, −18.5, −21, and −25 dB, respectively [44], and that the XT oscillation requires a −2 dB margin ($XT_{marg}$) [13]. Therefore, for a given modulation format *m*, the maximum transmission distance bounded by XT ($D_{m}^{XT}$) can be calculated by Equation (26):

(26) $$D_{m}^{XT}=max\left\{D|XT\left(D\right)\leq XT_{m}^{thre}+XT^{marg}\right\}$$

For connection requests in 4-core and 12-core MCFs with different modulation formats, we can calculate the maximum transmission distances bounded by OSNR ($D_{m}^{OSNR}$) [43] and XT ($D_{m}^{XT}$), respectively, as shown in Table 5. The maximum transmission distance is the small one of $D_{m}^{OSNR}$ and $D_{m}^{XT}$.

Therefore, the maximum transmission distances (km) of these modulation formats considered and the maximum traffic volume (Gbps) that an OC can carry under each modulation format are shown in Table 6.

The Gurobi optimizer v9.0.1 [45] is the solver software used to solve the models in this paper. Since the RSA problem is a well-known NP-hard problem that has been proven by [8,9], it is obviously NP-hard for the RMSSA problem that considers multiple spatial dimensions and modulation formats based on the RSA problem. Thus, for some instances, ILP models cannot be completely solved in a reasonable time (i.e., the obtained solution cannot be verified as optimal). To obtain a feasible solution of the model when solving each instance, we set an upper limit of 3600 s for the computation time of each part of the algorithms (e.g., in the DMD algorithm, the upper limit of computation time for the RMSA, SA, and RMSSA models are all 3600 s). The simulation experiments are executed in a Microsoft Windows 10 OS on a computer with an Intel 8-core 16-thread 3.6 GHz CPU and 64 GB memory.

### 4.2. Simulation Results of the DMD, ASLC-MD and SSLC-MD Algorithms

**Figure 7:**
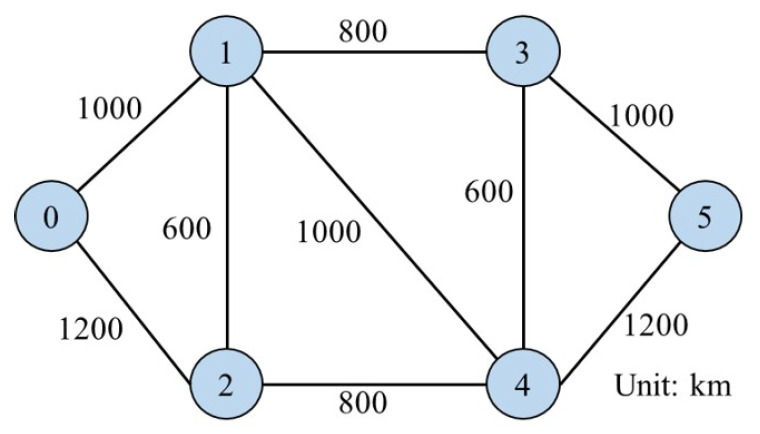
N6S9 network topology.

In this subsection, we compare the algorithms for solving the non-SLC node-type model in the N6S9 network shown in Figure 7, which consists of 6 nodes and 18 directed links interconnected by 4-core MCFs [37]. The available spatial switching granularities *i* are 1, 2, and 4, corresponding to the cases of Ind-Sw, FrJ-Sw, and J-Sw, respectively.

The traffic volume (Gbps) of each connection request is generated ranging from 100 Gbps to 1 Tbps in accordance with a uniform distribution. The source node and destination node of each connection request are randomly selected from the nodes in the network topology used. We consider different numbers of connection requests—from 50 to 200, 50 per step. Thirty traffic data sets are generated randomly for each number of connection requests.

Since there is not yet any work that involves multiple modulation formats by formulating node-type ILP models to solve the RMSSA problem, we consider lower bounds of the model and results of the following commonly used first-fit greedy (FF-G) algorithm [14] for solving the model as the indicators to evaluate the effectiveness of our algorithms. Regarding the FF-G algorithm as shown in Algorithm 1, SORTS is a set of different sorting methods. We discuss nine sorting methods according to the different properties of connection requests, which are sorting by traffic volume from small to large and large to small, the average hops of all paths from small to large and large to small, the hops of the shortest path from small to large and large to small, the hops of the longest path from small to large and large to small, and random sorting. We previously searched for all possible simple paths (i.e., paths without cycles) between the source and destination nodes of each connection request via the depth-first search (DFS) algorithm, and the set of all possible simple paths for the connection request *r* is denoted as $P_{r}$.
**Algorithm 1** First-fit greedy.1:Update the available FSs and spatial dimension groups2:**for**Method in SORTS **do**3:   Sort the set of connection requests *R* via Method4:   **for** *r* in *R* **do**5:     Find all physical paths $P_{r}$ for *r*6:     **for** *p* in $P_{r}$ **do**7:        Determine the most efficient modulation format $m_{r}^{p}$ for each physical path *p* via DAT8:     **end for**9:     Calculate $n_{r}^{m_{r}^{p}i}$10:   Create the candidate spectral blocks set $B_{r}$ with spatial continuity and FSs contiguity restrictions imposed, and $b_{r}^{mp}\in B_{r}$ is composed of $n_{r}^{m_{r}^{p}i}$ FSs11:   **for** $b_{r}^{mp}$ in $B_{r}$ **do**12:        Assign $b_{r}^{mp}$ to *r* on a trial and calculate the maximum index $FS_{max}^{b_{r}^{mp}}$ of the FSs used in the network after the trial assignment13:     **end for**14:     Find the minimum $FS_{max}^{b_{r}^{mp}}$, realign its corresponding $b_{r}^{mp}$ to *r* and update the available FSs and spatial dimension groups15:   **end for**16:   Record the maximum FSs index used in the network under the current sorting method of *R*17:**end for**18:Select the allocation scheme for the sorting method, which determines a minimum value of the maximum FSs index used in the network

Table 7 shows the experimental results for the maximum FS index of the non-SLC node-type model solved by the DMD, ASLC-MD, SSLC-MD and FF-G algorithms in the cases of spatial switching granularities *i* equal to 1, 2, and 4. The result is the value of the parameter ‘ObjVal’ output by Gurobi, which is the global optimal value when the model is completely solved in the time limit and is the local optimal value or current best value if a solution exists when the model is not completely solved in the time limit. ‘LB’ indicates the lower bound given by the parameter ‘Objbound’ of Gurobi when solving the RMSA model. The numbers listed in the parentheses indicate the numbers of sets in the 30 traffic data sets for which the optimal solutions are not obtained within the time limit (i.e., the number of times the model is not solved completely). The ASLC-MD algorithm does not obtain feasible solutions in the time limit for instances whose numbers of connection requests are 150 and 200. For the 30 traffic data sets with 150 connection requests, there are 24 sets when *i* is equal to 1 and 29 sets when *i* is equal to 2, where no feasible solution is found. For the 30 data sets with 150 connection requests, no feasible solutions are found for all 30 sets when *i* is equal to 1 and 2. As shown in Table 7, the results of the ASLC-MD algorithm are not very satisfactory, and it would be difficult to find feasible solutions in a reasonable time when the number of connection requests becomes larger. By comparing the results of the DMD, SSLC-MD, FF-G algorithms and lower bound, it can be observed that DMD and SSLC-MD can effectively solve the node-type model in the instances that were originally difficult to solve directly, and the qualities of the solutions obtained by DMD and SSLC-MD in the time limit were approximately the same and much better than the solutions obtained by heuristic FF-G algorithm.

Furthermore, it can be observed that with the number of connection requests being 150 and 200, all of our three algorithms yielded a large number of instances that could not be solved completely within the time limit. As we analyzed in Section 3.5, our model is strongly influenced by the number of connection requests. Therefore, when facing large-scale instances with a high number of connection requests, it will be difficult to solve in a reasonable time. In our pre-experiments, we noticed that the maximum number of connection requests that our algorithms can handle in the limited 10,000-second time is about 300. That is, for instances larger than 300 connection requests, we suggest using other efficient heuristic algorithms to solve them.

**Figure 8:**
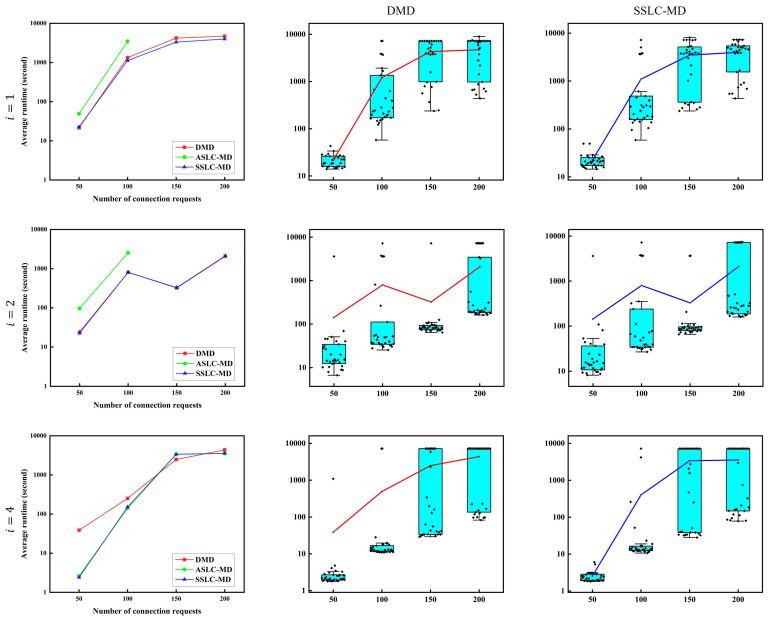
Execution time of the non-SLC node-type model solved by the DMD, ASLC-MD and SSLC-MD algorithms.

Figure 8 shows the experimental results regarding the execution time of the non-SLC node-type model solved by the DMD, ASLC-MD and SSLC-MD algorithms in the cases of spatial switching granularities *i* equal to 1, 2, and 4. The vertical coordinate called ‘average runtime’ indicates the average execution times of Gurobi when solving the model. The horizontal coordinate indicates the number of connection requests from 50 to 200 with 50 requests per step. Since the computation time of the heuristic FF-G algorithm is more than 10 times faster than those of the DMD, ASLC-MD, and SSLC-MD algorithms, which are used to solve the model, we do not include it in the comparison. The three figures in the left-hand column show the average execution times of the three algorithms used to solve the model for various values of spatial switching granularities *i*. The performance of the ASLC-MD algorithm was the worst. Moreover, by comparing the other two algorithms, DMD and SSLC-MD, it can be observed that in the case where the switching paradigms of the SLC and non-SLC models are different (i.e., the cases where i=1 and i=2), there is a larger number of spatial dimension groups, and the performance of SSLC-MD is superior to that of DMD (e.g., the average execution time of SSLC-MD is shorter than that of DMD in the case of i=1; however, in the case of i=2, since the number of spatial dimension groups is reduced, the advantage of SSLC-MD compared to DMD is also reduced). In the case where i=4, the switching paradigms of SLC and non-SLC are the same, and the results of DMD and SSLC-MD are both good and bad. The figures in the middle column and the right-hand column show the box plots of the results of DMD and SSLC-MD for various cases of spatial switching granularities *i*, respectively, and we can see the dispersion of the results for all 30 traffic data sets through these plots.

**Figure 9:**
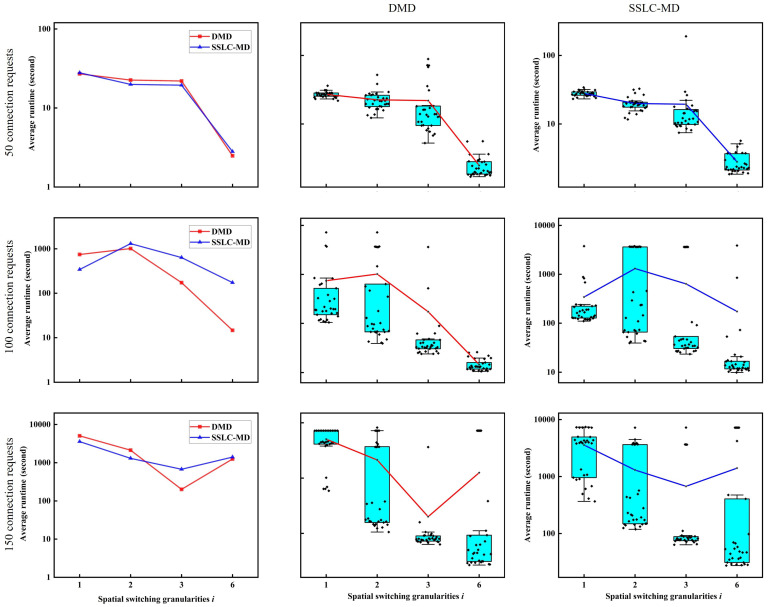
Execution time of the non-SLC node-type model solved by DMD and SSLC-MD algorithms in the case of the 6-core N6S9 network.

To better verify the difference in performance between the DMD and SSLC-MD algorithms with different numbers of spatial dimension groups, we increase the number of cores of MCF to 6 and perform experiments using the same 30 traffic data sets with connection request numbers of 50, 100, and 150. In this case, there are 4 possible values of spatial switching granularities, which are 1, 2, 3, and 6, corresponding to the numbers of spatial dimension groups, which are 6, 3, 2, and 1, respectively. Figure 9 shows the experimental results regarding the execution time of the non-SLC node-type model solved by the DMD and SSLC-MD algorithms in the cases of the 6-core N6S9 network. The figures in the middle column and the right hand column show the box plots of the results of DMD and SSLC-MD for various cases of spatial switching granularities *i*, respectively. It is obvious that when the spatial switching granularity *i* is small (i.e., when the number of spatial dimension groups is large, such as when i=1), the SSLC-MD algorithm has a shorter average computation time and shows better performance; however, when the spatial switching granularity *i* increases and the number of spatial dimension groups decreases, the SSLC-MD algorithm is inferior to the DMD algorithm. Therefore, to better solve the node-type model, we should select the appropriate decomposition algorithm according to the number of spatial dimension groups.

### 4.3. Comparison of the Proposed Non-SLC Node-Type Model and the Previous k-Path-Type Model

**Figure 10:**
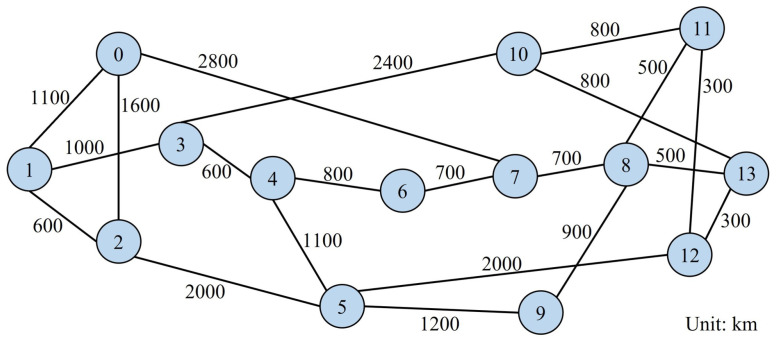
NSF network topology.

In this subsection, we compare the non-SLC node-type model (hereafter referred to as the node-type model) in this work with the previous k-path-type model in Ref. [11] (hereafter referred to as the k-path-type model) in the NSF network shown in Figure 10, which consists of 14 nodes and 42 directed links interconnected by 4-core MCFs. For illustrative purposes, we conduct simulation experiments only at Ind-Sw with spatial switching granularity i=1.

The set of candidate paths (k-shortest paths) for the k-path-type model is determined by the same routing algorithm as mentioned in Ref. [11]. We precompute two sets of candidate paths with candidate path numbers *K* equal to 2, 3, 4 and 5 in the NSF network and provide them to the k-path-type model. Since it takes a longer time to solve the node-type model, the node-type model is solved by using the solutions of the k-path-type model as the initial solutions. The upper limit of computation time for each model is set to 3600 s.

Table 8 and Table 9 show the results of the node-type model and the k-path-type model on the NSF network in our simulation. We performed simulation experiments for each of the four types of connection requests in the case where i=1 and the number of connection requests was equal to 100. ‘Random nodes’, ‘Same source nodes’, ‘Same destination nodes’ and ‘Not uniform nodes’ denote the types of connection requests whose source and destination are randomly generated, source nodes are the same, destination nodes are the same, and source and destination nodes are not all randomly generated, respectively (we fix the source and destination nodes of 20 of the 100 connection requests as nodes 4 and 9). Thirty traffic data sets are generated for each type of connection requests. The same instances of connection requests are solved for the two models. The numbers listed in the parentheses indicate the numbers of sets in the 30 traffic data sets for which the optimal solutions are not obtained within the time limit (i.e., the number of times the model is not solved completely). Results in Table 8 are output via the parameter ‘ObjVal’ of Gurobi and indicate the maximum FS index used in the network. The result is the optimal value when the model is completely solved in the time limit and is the feasible solution if a solution exists when the model is not completely solved in the time limit. As described in Section 4.2, the results in Table 9 indicate the lower bounds given by the parameter ‘Objbound’ of Gurobi. ‘Node’ and ‘K-Path’ indicate the node-type and k-path-type models, respectively.

We can observe that the qualities of the solutions of the k-path-type model are not better than those of the node-type one for the various types of connection requests. This is because the sets of candidate paths of the k-path-type model do not completely include all possible paths, which leads to the solutions obtained by the k-path-type model not being the optimal solutions. For instance, when K=2, the set of candidate paths for each connection request contains only two possible paths, so although the computation times for solving the model might be greatly reduced, the qualities of the solutions are more insecure. However, when K=3, the solutions (i.e., FS index) and the lower bound of the k-path-type model are improved compared to those of the case when K=2. We also observe that in the cases of K=4 and K=5, in some instances for the k-path-type model, although the lower bounds become closer to the lower bounds of the node-type model as *K* increases, the solutions become worse. The reason is that as *K* increases, the computational time required to solve the k-path-type model becomes much longer, so that more feasible but nonoptimal solutions that fail to solve the model completely appear. Therefore, it is often necessary to determine a reasonable value for the candidate path number *K* by pre-experimentation when we solve this type of problem with the k-path-type model. In addition, the node-type model is more difficult to solve in a short time than the k-path-type one, but since it considers all possible paths, if the node-type model can be solved completely, the qualities of its solutions are more reliable than those of the k-path-type one. On the other hand, the node-type model can be used to check the qualities of the solutions of k-path-type model for various values of *K* through operations such as solving the node-type model by using the solutions obtained by k-path-type model as the initial solutions (i.e., if the solutions obtained by the node-type model are better than those of k-path-type one, the solutions of k-path-type one can still be improved).

## 5. Conclusions

In this paper, we introduced the static resource allocation problem of SDM-EONs (i.e., the RMSSA problem) and proposed a node-type ILP model without SLC. Since this model has a large number of constraints and variables and is difficult to solve directly, we proposed three exact algorithms based on model decomposition (i.e., the DMD, ASLC-MD, and SSLC-MD algorithms) to better solve it. Through the comparison experiments, we investigated the performance of the DMD and SSLC-MD algorithms, which can solve the node-type model effectively, and found that we should select the appropriate decomposition algorithm according to the number of spatial dimension groups to better solve the node-type model. In addition, we also compared our node-type model with the k-path-type one in the previous work. The results indicate that our node-type model has an advantage over the previous k-path-type model in terms of solution quality, and our node-type model is a promising approach for checking the qualities of the solutions of the k-path-type model.

## Figures and Tables

**Table 1:** Related problems considered in previous works that formulated ILP models.

Refs.	OTN Type	Model		Spch Type			Modulation
		(k)-Path	Node	Spe SpCh	Spa SpCh	Spe & Spa SpCh	
[9,18,23,26]	EON	Y	N	Y	N	N	Y
[15,21]	EON	Y	Y	Y	N	N	N
[10,12]	EON	Y	Y	Y	N	N	Y
[13]	SDM-EON	Y	N	Y	N	N	Y
[28]	SDM-EON	Y	N	N	N	Y	Y
[29]	SDM-EON	Y	N	Y	N	Y	Y
[5]	SDM-EON	Y	Y	Y	N	N	N
[11,25]	SDM-EON	Y	N	Y	Y	Y	Y
our work	SDM-EON	N	Y	Y	Y	Y	Y

**Table 2:** List of parameters and variables.

Notations	Descriptions
Parameters:	
*i*	Spatial switching granularity
$G_{i}$	Set of spatial dimension groups g of spatial granularity *i*
*V*	Set of nodes in the network topology
*E*	Set of links in the network topology
$\sigma_{v}^{+}$	Set of links leaving from node v∈V
$\sigma_{v}^{-}$	Set of links entering node v∈V
$l^{e}$	Length of link e∈E
MF	Set of available modulation levels
$a^{m}$	Maximum transmission distance allowed by modulation level m∈MF
*R*	Set of connection requests
$s_{r}$	Source node for connection request r∈R
$d_{r}$	Destination node for connection request r∈R
$T_{r}$	Traffic volume [Gbps] of connection request r∈R
$t_{OC}$	Traffic volume [Gbps] that a single OC using the modulation format with the lowest spectral efficiency can support
$W_{FS}$	Bandwidths [GHz] occupied by a FS
$W_{OC}$	Bandwidths [GHz] occupied by a single OC
$W_{GB}$	Bandwidths [GHz] occupied by a switching GB
$n_{r}^{mi}$	Number of FSs required for transmitting connection request r∈R by using modulation level m∈MF in spatial switching granularity *i*
$m_{r}^{out}$	Selected modulation level for serving connection request *r* obtained by solving the RMSA model
*M*	A value that is large enough
Variables:	
$x_{r}^{eg}\in \left\{0,1\right\}$	A binary variable that is equal to 1 if connection request *r* uses spatial dimension group *g* on link *e* and 0 otherwise
$u_{r}^{m}\in \left\{0,1\right\}$	A binary variable that is equal to 1 if connection request *r* uses the modulation level *m* and 0 otherwise
$o_{rr^{\prime }}\in \left\{0,1\right\}$	A binary variable that is equal to 1 if the index of starting FS used by *r* is smaller than that used by $r^{\prime }$ and 0 otherwise
$b_{r}^{meg}\in \left\{0,1\right\}$	A binary variable that is equal to 1 if connection request *r* uses modulation level *m* and spatial dimension group *g* on link *e* and 0 otherwise
$h_{r}\in R_{+}$	A nonnegative continuous variable that represents the length of the path used by connection request *r*
$f_{r}\in Z_{+}$	A nonnegative integer variable that indicates the index of the first FS used by the connection request *r*
$F_{max}\in Z_{+}$	A nonnegative integer variable that indicates the maximum index of required FSs in the network topology
$F_{max}^{lb}\in Z_{+}$	A nonnegative integer variable that indicates the maximum index of required FSs in the RMSA model
$F_{max}^{ub}\in Z_{+}$	A nonnegative integer variable that indicates the maximum index of required FSs in the SA model

**Table 3:** Number of variables and constraints per model.

Models	Variables	Constraints
RMSSA	$O(|R|\cdot |E|\cdot |G_{i}\left|+{\left|R\right|}^{2}\right)$	$O(|R|\cdot |V|\cdot |G_{i}|+{\left|R\right|}^{2}\cdot |E|\cdot |G_{i}\left|\right)$
SLC-RMSSA	$O(|R|\cdot |E|\cdot |G_{i}\left|+{\left|R\right|}^{2}\right)$	$O(|R|\cdot \left|V\right|+{\left|R\right|}^{2}\cdot |E|\cdot |G_{i}\left|\right)$
RMSA	$O(|R|\cdot |MF|\cdot |E|\cdot |G_{i}\left|\right)$	$O(|R|\cdot |V|\cdot |G_{i}\left|+|R|\cdot |MF|\cdot |E|\cdot \right|G_{i}\left|\right)$
SLC-RMSA	$O(|R|\cdot |MF|\cdot |E|\cdot |G_{i}\left|\right)$	$O(|R|\cdot |V|+|R|\cdot |MF|\cdot |E|\cdot |G_{i}\left|\right)$
SA	$O({\left|R\right|}^{2})$	$O({\left|R\right|}^{2})$

**Table 4:** Physical features of the MCFs considered in the simulation experiments.

Fiber Type	*k*	Λ	β	γ
4-core MCF [37]	$5.0\times 10^{-4}$	$3.9\times 10^{-5}$	$4.0\times 10^{6}$	$5.0\times 10^{-2}$
12-core MCF [38]	$1.4\times 10^{-3}$	$3.7\times 10^{-5}$	–	–

**Table 5:** Maximum transmission distances bounded by OSNR and XT in 4-core MCFs and 12-core MCFs under different modulation formats *m*.

Limitation Factor	Maximum Transmission Distance (km)			
	DP-BPSK	DP-QPSK	DP-8QAM	DP-16QAM
$D_{m}^{OSNR}$ [43]	6300	3500	1200	600
$D_{m}^{XT}$ in 4-core MCFs	38,945	13,872	7808	3111
$D_{m}^{XT}$ in 12-core MCFs	4712	1678	944	376

**Table 6:** Maximum transmission distance of each modulation format and maximum traffic volume supported of each OC fixed at 32 Gbaud.

Modulation Format	DP-BPSK	DP-QPSK	DP-8QAM	DP-16QAM
Transmission distance (km) in 4-core MCFs	6300	3500	1200	600
Transmission distance (km) in 12-core MCFs	4712	1678	944	376
Traffic volume per OC (Gbps)	50	100	150	200

**Table 7:** Maximum FS index of the non-SLC node-type model solved by the DMD, ASLC-MD, SSLC-MD and FF-G algorithms.

|R|	*i*	Algorithms				LB
		DMD	ASLC-MD	SSLC-MD	FF-G	
50	1	30.60 (0)	30.60 (0)	30.60 (0)	31.80 (0)	30.60 (0)
	2	30.55 (0)	30.55 (0)	30.55 (0)	33.33 (0)	30.55 (0)
	4	37.87 (0)	37.87 (0)	37.87 (0)	41.40 (0)	37.87 (0)
100	1	49.76 (5)	50.60 (14)	49.68 (5)	53.53 (0)	49.28 (1)
	2	55.90 (3)	56.25 (8)	55.80 (3)	59.63 (0)	55.55 (1)
	4	69.63 (2)	69.57 (1)	69.60 (2)	75.73 (0)	69.50 (0)
150	1	74.93 (19)	–	74.52 (17)	76.53 (0)	70.90 (4)
	2	80.20 (1)	–	80.57 (2)	86.30 (0)	80.17 (0)
	4	102.50 (9)	102.63 (13)	102.60 (13)	110.63 (0)	102.17 (0)
200	1	95.03 (19)	–	96.43 (21)	99.30 (0)	92.07 (4)
	2	105.67 (7)	–	105.70 (8)	112.80 (0)	105.33 (0)
	4	134.83 (18)	134.67 (14)	134.67 (14)	144.17 (0)	133.60 (0)

**Table 8:** Comparison of the maximum FS index used in the network for node-type and k-path-type models.

Types of Connection Requests	Node	K-Path			
		K=2	K=3	K=4	K=5
Random nodes	59.23 (7)	60.13 (3)	59.30 (4)	59.50 (4)	59.50 (4)
Same source nodes	120.93 (7)	138.67 (0)	121.13 (0)	121.60 (3)	122.67 (7)
Same destination nodes	120.67 (15)	126.30 (1)	120.77 (2)	122.00 (5)	121.20 (7)
Not uniform nodes	66.00 (16)	72.30 (8)	68.47 (13)	68.13 (18)	66.57 (7)

**Table 9:** Comparison of the lower bounds for node-type and k-path-type models.

Types of Connection Requests	Node	K-Path			
		K=2	K=3	K=4	K=5
Random nodes	59.03 (2)	60.03 (0)	59.17 (0)	59.10 (0)	59.07 (0)
Same source nodes	120.93 (0)	138.67 (0)	121.13 (0)	120.93 (0)	120.93 (0)
Same destination nodes	120.50 (0)	126.27 (0)	120.70 (0)	120.50 (0)	120.50 (0)
Not uniform nodes	65.47 (10)	72.03 (0)	67.83 (0)	66.93 (0)	65.83 (0)

## Data Availability

For the data of network frameworks, please refer to [11,12,36]. For data of the MCFs, please refer to [37,38].
